# The Zn- or Cu-Thionein Character of a Metallothionein Determines Its Metal Load When Synthesized in Physiological (Metal-Unsupplemented) Conditions

**DOI:** 10.1155/2010/541829

**Published:** 2010-05-05

**Authors:** Mercè Capdevila, Òscar Palacios, Sílvia Atrian

**Affiliations:** ^1^Departament de Química, Facultat de Ciències, Universitat Autònoma de Barcelona, 08193 Cerdanyola del Vallès (Barcelona), Spain; ^2^Departament de Genètica, Facultat de Biologia, Universitat de Barcelona, Institut de Biomedicina de la Universitat de Barcelona (IBUB), 08028 Barcelona, Spain

## Abstract

The present work comprises the recombinant synthesis of four metallothioneins (MTs) in metal-unsupplemented cultures and the characterization of the recovered metal complexes by means of analytical and spectrometric techniques. The four MTs are two *Drosophila* (MtnA and MtnB), one yeast (Crs5), and one mouse (mMT1) metallothionein isoforms. These four MTs exhibit distinct metal binding preferences, from a clear Cu-thionein character to a definite Zn-thionein nature, respectively. Although in all cases, the only metal ion present in the purified complexes is Zn^2+^, our results highlight an inherently different behaviour of those two types of MTs, in conditions that would mimic their synthesis in physiological environments. Therefore, intrinsically different roles can be hypothesized for the constitutively-produced MT peptides in the absence of any metal overload, depending on their Zn- or Cu-thionein character.

## 1. Introduction

Metallothioneins (MTs) are a superfamily of small proteins, ubiquitous and probably polyphyletic, which coordinate heavy-metal ions through metal-thiolate bonds established by their highly abundant cysteine residues [1]. Currently, and since they were first reported in 1957 [2], MTs biological structure and their contribution to a variety of physiological processes in the most diverse organisms still remain a matter of debate [3]. Besides their detoxifying properties, MTs appear to be involved in Zn and Cu homeostasis, while additionally they seem to participate in a myriad of processes specific to each different group of organisms considered. 

 Apo-MTs (also called thioneins) are random coil polypeptides, which only fold onto a definite 3D structure upon metal coordination. This implies that MTs adopt different 3D structures—the main determinant of protein functionality-depending on the nature and number of the metal ions that they coordinate in a given metal complex. This is especially noteworthy because although the level of expression of *MT* genes remains generally low in the absence of metal induction, the possibility exists that some organisms or tissues accumulate demetalated (without metal ions) thionein peptides under definite conditions. In fact, some years ago, it was claimed that the levels of thionein in mammalian tissues under physiological conditions could be significantly high enough to harbour important implications for MT functionality in zinc and redox metabolism [4, 5]. However, native MT synthesis is extremely low unless the corresponding gene is induced by the presence of a specific metal ion or other stress conditions. This, in turn, practically precludes the purification of native apo-forms, produced in physiological conditions. This serious drawback can be avoided by means of recombinant synthesis. Remarkably, although this strategy has been widely used by many authors to obtain metal-MT complexes of the most diverse organisms, it has seldom been used to study MT synthesis in physiological environments in the absence of metal surplus. Several years ago we set up an *E. coli * expression system that enabled the biosynthesis of Zn^2+^-, Cd^2+^-, and Cu^+^-MT complexes, equivalent to those natively synthesized, of sufficient quantity and purity to permit analytical, spectrometric, and spectroscopic characterization, by growing the corresponding recombinant bacteria in metal supplemented media [6, 7]. In this work, we have adapted the same synthesis rationale to study the metal binding abilities of different MTs when produced in bacteria grown in standard (i.e., nonmetal supplemented media), particularly in order to determine the features of the zinc-complexes biosynthesized in these conditions. 

 To perform this study we selected four Metallothioneins, belonging to a broad spectrum of organisms: two *Drosophila* (MtnA and MtnB), a yeast (Crs5), and a mouse (mMT1) isoform (Table 1), because they exhibit distinct metal binding preferences when considered in accordance with our classification as Zn-thionein versus Cu-thionein [8]. We have recently revised this first classification scheme, based on two discrete MT groups, and we now propose a stepwise gradation between extreme Zn-thioneins and Cu-thioneins that holds a continuum of intermediate forms in terms of metal binding properties [9]. The results we include in this paper clearly demonstrate, first, that all MTs are able to some extent to capture and coordinate Zn^2+^ ions present in standard (nonsupplemented) bacterial growth medium (LB). But, secondly, it worths noting how our results show that the average zinc content and the precise Zn-MT complexes recovered for a given MT peptide depend on its situation in our gradate classification, in other words, on its Zn- versus Cu-thionein character. Therefore, on the one hand this work sheds light on the behaviour of MT polypeptides when there is no metal surplus in the surrounding environment; and on the other hand, it adds a new criterion to support our classification of MTs according to their metal handling abilities.

## 2. Experimental

### 2.1. Synthesis of the Recombinant Metal-MT Complexes

Recombinant syntheses of MTs (primarily as GST-MT fusions) were achieved through expression of the corresponding cDNAs cloned in pGEX vectors. Construction of these plasmids is reported in detail in [7] for the mouse MT1 isoform, [10] for the yeast *S. cerevisiae* Crs5 MT, and [11, 12] for the *D. melanogaster* Mtn (or MtnA) and Mto (or MtnB) isoforms, respectively. All the metal-MT complexes analyzed in this work were purified from 1L cultures of BL21 *E. coli *cells transformed with the corresponding pGEX-MT plasmid, grown in standard LB (Luria-Bertani) broth with 100 mg mL^−1^ ampicillin and no metal supplement, except for the control condition, when 300 *μ*M ZnCl_2_ was added to the LB medium. GST-MT synthesis was induced with isopropyl-1-thio-*β*-D-galactopyranoside (IPTG) at a final concentration of 100 mM. After 2.5-hour induction, cells were harvested by centrifugation. To prevent oxidation of the metal-MT complexes, argon was bubbled in all the steps of the purification following cell disruption. For protein purification, cells were resuspended in ice-cold PBS (1.4 M NaCl, 27 mM KCl, 101 mM Na_2_HPO_4_, and 18 mM KH_2_PO_4_) 0.5% v/v *β*-mercaptoethanol, disrupted by sonication, and centrifuged at 12,000 g for 30 minutes. The GST-MT fusions were purified from the recovered supernatant by gluthatione-Sepharose 4B (GE Healthcare) batch affinity chromatography incubating the mixture with gentle agitation for 60 minutes at room temperature. After three washes in PBS, and due to the fact that the GST-MT constructs include a thrombin recognition site, this protease was added (10 u per mg of protein) and digestion was carried out overnight at 23–25°C. This allowed separation of the GST portion—which remained bound to the gel matrix—from the MT moiety (that eluted together with thrombin). Subsequently, this eluate was concentrated using Centriprep Concentrators (Amicon) (Millipore, Billerica, MA, USA) with a cut-off of 3 kDa and fractionated using FPLC, through a Superdex-75 column (GE Healthcare) equilibrated with 50 mM Tris-HCl, pH 7.0, and run at 1 mL min^−1^. Fractions were collected, and analysed for protein content by their absorbance at 254 nm. Aliquots of the protein-containing FPLC fractions were analysed by 15% SDS-PAGE and stained by Coomassie Blue. MT-containing samples were pooled and stored at −70°C until further characterization. 

### 2.2. Inductively Coupled Plasma Atomic Emission Spectroscopy (ICP-AES)

The recombinantly expressed MT complexes were analyzed for element composition (S, Zn, Cd and, Cu) by inductively coupled plasma atomic emission spectroscopy (ICP-AES) on a Polyscan 61E spectrometer (Thermo Jarrell Ash Corporation, Franklin, MA, USA) at appropriate wavelengths (S, 182.040 nm; Zn, 213.856 nm; Cd, 228.802 nm; Cu, 324.803 nm). Samples were prepared either at “conventional” (dilution with 2% HNO_3_ (v/v)) [13] or at “acidic” (incubation in 1 M HCl at 65°C for 5 minutes) conditions [14]. MTs concentration in the recombinant preparations was calculated assuming that the only contribution to their S content was that made by the MT peptides.

### 2.3. Mass Spectrometry

Molecular mass determination was performed by electrospray ionization mass spectrometry equipped with a time-of-fly analyzer (ESI-TOF MS) using a Micro Tof-Q Instrument (Brucker Daltonics Gmbh, Bremen, Germany) calibrated with NaI (200 ppm NaI in a 1 : 1 H_2_O:isopropanol mixture), interfaced with a Series 1100 HPLC pump (Agilent Technologies) equipped with an autosampler, both controlled by the Compass Software. The experimental conditions for analyzing MT samples were: 20 *μ*L of the sample was injected through a PEEK long tube (1.5 m × 0.18 mm i.d.) at 40 *μ*L/min under the following conditions: capillary-counterelectrode voltage, 5.0 kV; desolvation temperature, 90–110°C; dry gas, 6 L/min. Spectra were collected throughout an m/z range from 800 to 2000. The liquid carrier was a 90 : 10 mixture of 15 mM ammonium acetate and acetonitrile, pH 7.0. All the samples were injected at least in duplicate to ensure reproducibility. In all cases, molecular masses were calculated according to the reported method [15].

## 3. Results and Discussion

The four MTs studied in this work (Table 1) cover a broad spectrum of organisms: from vertebrates—the mouse mMT1 peptide—to invertebrates—two *Drosophila* (MtnA and MtnB) peptides—and, unicellular eukaryotes—the yeast Crs5 MT. Besides, they exhibit differential metal binding abilities towards Zn^2+^ and Cu^+^, as evidenced by their position in our Zn- versus Cu-thionein gradated classification [9]. The overall results of this study are presented in a comprehensive table (Table 2) for the sake of easy comparison, but they are discussed independently below. One of the MTs, Mtn-B, was also synthesised under excess zinc conditions (300 *μ*M in the culture medium), as a positive control in relation to the nonsupplemented experiments. It is extremely important to highlight that in all cases, the Cd and Cu content of the preparations was also measured, but the obtained values were always not significant. This is consistent with the metal ion content reported for the intracellular environment of *E. coli* cells, which has been estimated to be about 0.1 mM for zinc while only in the 10-to-100 *μ*M range for copper [16], so Zn^2+^ ions are indeed the only ones available for the nascent MT peptides. 

**Figure 1:**
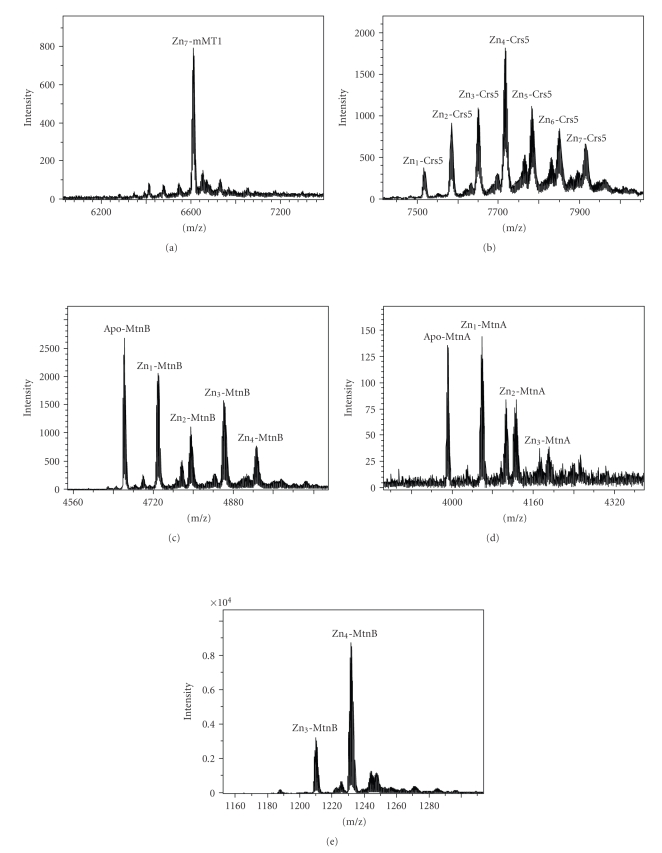
Deconvoluted ESI-TOF MS spectra of the recombinant Zn(II)-MT complexes obtained from the pGEX-MT expression system with no zinc supplementation to the culture: (a) mouse MT1; (b) yeast Crs5; (c) *D. melanogaster* MtnB and (d) *D. melanogaster* MtnA. (e) MtnB was also synthesized in zinc-enriched medium, to be used as a control.

 The mMT1 isoform, one of the four encoded in the mouse (mammalian) genomes, has for a long time been the paradigm of divalent-metal binding MTs. Accordingly, it is in the third position out of sixteen in our gradate classification of Zn-thioneins, as shown in Table 3. This means that the corresponding polypeptide has a high ability to form complexes with divalent metal ions (Zn^2+^ and Cd^2+^) so that these complexes are well folded and remain steady in physiological conditions. Strikingly, recombinant synthesis of mMT1 renders unique Zn_7_-mMT1 complexes (Figure 1(a), Table 2), regardless of whether the bacterial culture has been supplemented with zinc (300 *μ*M) [7] or not. This suggests an extreme proficiency of mMT1 not only to form well-structured Zn-complexes when these metal ions are in surplus, but also to capture the Zn^2+^ ions present in a standard environment, which may be of significant importance when considering its potential functions in physiological conditions. 

 Crs5 is a *Saccharomyces cerevisiae* MT, nonhomologous to the paradigmatic Cu-thionein Cup1 present in the same yeast species. Despite initially being considered a secondary copper-resistance agent, we showed that it determines survival under zinc overload, and furthermore, all the data concerning its metal-binding abilities converged to define the partial Zn-thionein character of Crs5 [10]. In fact, it is located in a rather intermediate position in our Zn- versus Cu-thionein ranking (10th position out of 16 MTs, Table 3). When this MT is synthesized in the absence of Zn overload, not only does the mean Zn-per-MT ratio diminish from 5.7 to 4.2 (Table 2), but there is also a huge variety of Zn-Crs5 species produced, as revealed by the ESI-MS spectrum of the sample (Figure 1(b)). Besides the major Zn_4_-Crs5 species, the preparation included other complexes, exhibiting a wide range of stoichiometries, from the highest value observed under zinc supplementation (Zn_7_-Crs) to the major species recovered then [10] (Zn_6_- and Zn_5_-Crs5), as well as other undermetalated (without metals) complexes, from Zn_3_- to Zn_1_-Crs5. It is therefore clear that this MT is able to coordinate some zinc ions from normal physiological environments, but in this case, more than half of the polypeptides encompass a zinc load lower than they would in excess zinc conditions.

 Finally, the two *Drosophila* MTs, MtnA and MtnB, served to study the behaviour of Cu-thioneins, since they have been characterized as the animal MTs that are most similar to the paradigmatic Cup1 Cu-thionein [9]. Consequently both polypeptides have been able to render homonuclear Cu(I)-complexes upon recombinant synthesis in copper-supplemented media [11, 12]. Despite these properties, Cu-thioneins are able to fold into Zn^2+^-containing complexes when produced under excess zinc as observed in the control experiment (Figure 1(e), Table 2) and literature data [11, 12]. However, our current results provide evidence that this is not the case in nonsupplemented media, since both MtnA and MtnB are recovered mostly as apo-forms or Zn_1_-Mtn complexes (Table 2). MS spectra of these preparations (Figures 1(c) and 1(d)) reveal that both MT polypeptides are severely undermetalated (without metals), with a high abundance of complexes exhibiting a metal content far below that they can attain as fully loaded species (Table 3). Therefore, it has to be assumed that should these MTs be present in a normal environment (i.e., with no metal ion surplus), they would coordinate very few Zn^2+^ ions, if any. 

 The joint observation of these results leads to important considerations. First, it is obvious that the levels of Zn(II) concentrations normally present in the cell cytoplasm (estimated in 0.1 mM for *E. coli *[16]) enable the building of Zn-MT complexes, even in the absence of zinc overload. This is extremely important for predicting the fate of the native MT peptides translated in uninduced conditions, and it would be in full agreement with the hypothesis that in basal conditions, MTs exhibiting a clear Zn-thionein character are synthesized inside cells as Zn^2+^-complexes, which undergo the corresponding exchange reaction in the presence of an excess of other metal ions [17]. However, the Zn-MT species yielded by the synthesis in nonsupplemented media, and their relation with those obtained from conventional zinc-supplemented cultures, are highly dependent on the type of MT considered, and precisely, on its Zn-thionein character. Hence, the mouse MT1 isoform, a genuine Zn-thionein, yields exactly the same result in both culture conditions, showing its extreme ability to capture Zn^2+^ ions from its physiological environment. On the other hand, the genuine Cu-thioneins analyzed are almost unable to yield Zn^2+^-containing complexes unless they are synthesized under excess zinc, even rendering apo-forms or Zn_1_-MT as major products (cf. results for MtnA and MtnB, Figure 1, Table 2). Comparison of the synthesis in normal and zinc-enriched media (Table 3), both regarding the mean Zn : MT content ratio and the Zn^2+^-containing species recovered, highlights that the higher the Zn-thionein character of a MT peptide, the more similar will be the results of the synthesis of Zn-MT complexes in both conditions. And, on the contrary, the greater the Cu-thionein character, the more different will be the species resulting from these syntheses, to the point that Cu-thioneins are characterized by an inherent incapacity to coordinate Zn^2+^ ions when present at physiological concentrations. This means that Cu-thioneins, if synthesized as the result of the constitutive expression of their genes (i.e., under no metal surplus), would remain mainly as apo-peptides inside cells, but never as basal Zn^2+^-containing complexes.

## 4. Conclusion

Comprehensive consideration of our data suggests that the result of synthesizing different MTs in nonmetal-supplemented media is highly dependent on their Zn- versus Cu-thionein character. Although in all cases Zn^2+^-containing complexes are produced, the former practically yields the same Zn-MT species when recombinantly produced in zinc-supplemented and in normal (nonsupplemented) medium, while the latter is almost unable to coordinate Zn^2+^ unless these metal ions are in clear surplus (300 *μ*M in culture media). These results may reflect an intrinsically different role of these two types of MTs in physiological conditions, as well as their well-assumed different role when they are natively synthesized as a protective response to the corresponding cognate-metal overload.

## Figures and Tables

**Table 1:** Amino acid sequences of the four MT isoforms analyzed in this work. MtnA and MtnB are two *Drosophila melanogaster* isoforms. Crs5 is a *Saccharomyces cerevisiae* MT and mMT1 is the mouse MT isoform 1. The GST-based expression system used for recombinant synthesis added two N-terminal residues to these sequences (GS), which was previously shown not to influence the binding properties of MT peptides [7], but it has to be considered for the calculation of their theoretical molecular masses (Table 2, Figure 1).

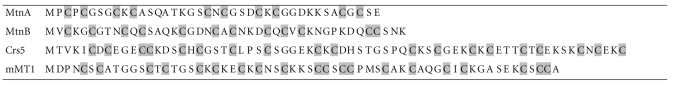

**Table 2:** Metal-to-protein ratios and molecular masses of the recombinant Zn(II)-MT, complexes obtained from the pGEX expression system with no metal supplementation to the culture, except in the Zn-MtnB used as a control (see Section 2). All the measurements have been performed at least twice, from independently obtained samples.

MT protein	Zn/MT^(a)^	Zn/MT^(a)^	Zn-MT species^(b)^	*m*_exp _^(c)^	*m*_th_^(d)^
	(ICP-AES)	(acidic ICP-AES)	(ESI-MS)		
mMT1	6.95	7.40	**Zn_7_-mMT1**	6605.5	6605.96

Crs5	4.20	4.61	**Zn_4_-Crs5**	7715.5	7718.92
			Zn_3_-Crs5 · Zn_5_-Crs5	7654.5 · 7780.5	7655.53 · 7782.31
			Zn_2_-Crs5 · Zn_6_-Crs5	7590.4 · 7844.5	7592.13 · 7845.70
			Zn_1_-Crs5 · Zn_7_-Crs5	7525.0 · 7907.5	7528.75 · 7909.09

MtnB	2.04	2.40	**apo-MtnB**	4667.2	4669.34
			Zn_1_-MtnB	4733.8	4732.73
			Zn_3_-MtnB	4857.0	4859.51
			Zn_2_-MtnB	4795.0	4796.12
			Zn_4_-MtnB	4920.8	4922.9

MtnA	1.70	1.94	**apo-MtnA **·** Zn_1_-MtnA**	3996.4 · 4059.4	3997.49 · 4060.88
			Zn_2_-MtnA	4121.2	4124.27
			Zn_3_-MtnA	4186.4	4187.66

Zn-MtnB^(e)^	3.95	5.10	**Zn_4_-MtnB**	4922.0	4922.90
			Zn_3_-MtnB	4850.2	4859.51

*ICP-AES* inductively coupled plasma atomic emission spectroscopy, *ESI-MS* electrospray ionization mass spectrometry.

^(a)^In all experiments Cd and Cu contents were also measured but their amount was always under detection limits.

^(b)^Species proposed according to the mass difference between holo-protein and apo-protein. Species in bold are the major components of the preparation.

^(c)^Experimental molecular masses. Measurements were always performed in duplicate. All corresponding standard deviations were always less than 1%.

^(d)^Theoretical molecular mass of the corresponding species.

^(e)^Control experiment: MtnB synthesized in Zn(II)-supplemented medium.

**Table 3:** Comparison of the Zn-MT complexes recovered from recombinant syntheses in Zn- and nonsupplemented culture media.

MT protein	Zn-MT species recovered when synthesized in Zn-supplemented media	Zn-MT species recovered when synthesized in nonsupplemented media^(a)^	Mean Zn : MT molar ratio, when synthesized in Zn-supplemented media	Mean Zn : MT molar ratio, when synthesized in nonsupplemented media^(b)^	Situation in the Zn-/Cu-thionein gradation^(c)^
mMT1	Zn_7_-mMT1 [7]	Zn_7_-mMT1	7.3 [7]	6.95	3/16
Crs5	Zn_6_-Crs5 · Zn_5_-Crs5 [10]	Zn_4_-Crs5	5.7 [10]	4.20	10/16
MtnA	Zn_4_-MtnA [11]	Apo-MtnA · Zn_1_-MtnA	3.5 [11]	1.70	14/16
MtnB	Zn_4_-MtnB [12]	Apo-MtnB	3.7 [12]	2.04	15/16

^(a)^In this work, only major species identified by ESI-MS are indicated.

^(b)^In this work, the mean Zn : MT value corresponding to the conventional ICP-AES value is indicated.

^(c)^The study in [9] includes 16 MT isoforms, classified from the most extreme Zn-thionein to the most extreme Cu-thionein character.
